# Biomedical journal speed and efficiency: a cross-sectional pilot survey of author experiences

**DOI:** 10.1186/s41073-017-0045-8

**Published:** 2018-01-05

**Authors:** Joshua D. Wallach, Alexander C. Egilman, Anand D. Gopal, Nishwant Swami, Harlan M. Krumholz, Joseph S. Ross

**Affiliations:** 1 Center for Outcomes Research and Evaluation, Yale–New Haven Hospital, 1 Church Street, Suite 200, New Haven, CT 06520 USA; 2 Collaboration for Research Integrity and Transparency, Yale Law School, 157 Church Street, 17th Floor, Suite 1, New Haven, CT 06520 USA; 3 Yale University School of Medicine, New Haven, CT USA; 4 Yale University, New Haven, CT USA; 5 Section of Cardiovascular Medicine and National Clinician Scholars Program, Department of Internal Medicine, Yale School of Medicine, New Haven, CT USA; 6 Department of Health Policy and Management, Yale School of Public Health, New Haven, CT USA; 7 Section of General Medicine and the National Clinician Scholars Program, Department of Internal Medicine, Yale School of Medicine, New Haven, CT USA

**Keywords:** Peer-review, Publication process, Pre-prints

## Abstract

**Background:**

Although the peer review process is believed to ensure scientific rigor, enhance research quality, and improve manuscript clarity, many investigators are concerned that the process is too slow, too expensive, too unreliable, and too static. In this feasibility study, we sought to survey corresponding authors of recently published clinical research studies on the speed and efficiency of the publication process.

**Methods:**

Web-based survey of corresponding authors of a 20% random sample of clinical research studies in MEDLINE-indexed journals with Ovid MEDLINE entry dates between December 1 and 15, 2016. Survey addressed perceived manuscript importance before first submission, approximate first submission and final acceptance dates, and total number of journal submissions, external peer reviews, external peer reviewers, and revisions requested, as well as whether authors would have considered publicly sharing their manuscript on an online platform instead of submitting to a peer-reviewed journal.

**Results:**

Of 1780 surveys distributed, 27 corresponding authors opted out or requested that we stop emailing them and 149 emails failed (e.g., emails that bounced *n* = 64, returned with an away from office message *n* = 70, or were changed/incorrect *n* = 15), leaving 1604 respondents, of which 337 completed the survey (21.0%). Respondents and non-respondents were similar with respect to study type and publication journals’ impact factor, although non-respondent authors had more publications (*p* = 0.03). Among respondents, the median impact factor of the publications’ journal was 2.7 (interquartile range (IQR), 2.0–3.6) and corresponding authors’ median h-index and number of publications was 9 (IQR, 3–20) and 27 (IQR, 10–77), respectively. The median time from first submission to journal acceptance and publication was 5 months (IQR, 3–8) and 7 months (IQR, 5–12), respectively. Most respondents (62.0%, *n* = 209) rated the importance of their research as a 4 or 5 (5-point scale) prior to submission. Median number of journal submissions was 1 (IQR, 1–2), external peer reviews was 1 (IQR, 1–2), external peer reviewers was 3 (IQR, 2–4), and revisions requested was 1 (IQR, 1–1). Sharing manuscripts to a public online platform, instead of submitting to a peer-reviewed journal, would have been considered by 55.2% (*n* = 186) of respondents.

**Conclusion:**

Corresponding authors have high perceptions of their research and reported requiring few manuscript submissions prior to journal acceptance, most commonly by lower impact factor journals.

**Electronic supplementary material:**

The online version of this article (10.1186/s41073-017-0045-8) contains supplementary material, which is available to authorized users.

## Background

In the scientific community, there is enormous pressure to publish as often and as quickly as possible––preferably in the highest impact peer-reviewed journals. The publication of a scientific manuscript is often considered the final stage of the research process, initiated once a project is complete and ready for peer judgment; it involves manuscript preparation, peer review, revision, and in many cases, re-submission to multiple journals before acceptance. Although the peer review process is believed to ensure scientific rigor, enhance research quality, and improve manuscript clarity, many investigators are concerned that the process is too slow [1, 2], too expensive, too unreliable, and too static [3–6].

Although there is a growing interest among the scientific community in increasing value and reducing waste in biomedical research [7], little empirical data exist on author experiences when it comes to the speed and efficiency of the publication process. Previous evaluations have focused on author perspectives and experiences submitting their work to one particular journal [4, 6]. While it is possible to collect information on the peer review process from individual journals, including the average number of days from submission of a manuscript to first decision, these data do not reflect the scientists’ perception of the broader publication process, which may involve multiple submissions to multiple different journals and long perceived delays from completion of the work to final dissemination to the peer community of researchers.

In this feasibility study, our objective was to pilot a survey of corresponding authors of recently published clinical research studies in order to quantify the speed and efficiency of the broader publication process, including the history of prior submissions and reviews and the time from first submission to final publication. We were specifically interested in whether a diverse set of corresponding authors of clinical research studies would respond to an online survey in sufficient numbers.

## Methods

We conducted a cross-sectional survey of a sample of investigators who published clinical research studies in MEDLINE-indexed biomedical journals with Ovid MEDLINE entry dates between December 1, 2016 and December 15, 2016. These entry dates were selected to help minimize any recall bias and to ensure that the author contact information provided in the manuscript was up-to-date. MEDLINE searches were conducted through Ovid (Ovid Technologies, New York). After mapping the tree for the term “Clinical Study,” we selected the terms “Clinical Trial,” “Observational Study,” and “Meta-Analysis.” Additional custom limits and specific search terms for observational studies are included in the Additional file 1.

### Study sample and design

We randomly selected a 20% sample of the 10,631 articles identified through the Ovid MEDLINE search. Two investigators (JDW, ACE) reviewed the titles and abstracts of all potential articles to exclude all non-clinical and non-research publications (i.e., letters, viewpoints).

### Data extraction

Information cataloged routinely by Ovid MEDLINE was used to determine the journal name, author(s) names, DOI number, date and country of publication, and title. Based on the abstract and/or introduction, each eligible article was then classified as an *observational or other study*, *clinical trial*, or *systematic review and/or meta-analysis* (Table 1). The first corresponding author named in each article was identified for participation. When the same corresponding author was listed in multiple articles, we randomly selected one publication for inclusion. When full-text articles did not provide an email address for the corresponding author, additional articles listed in the Scopus database for the same corresponding author were reviewed. To determine the 2015 impact factor of each publication’s journal, four investigators (JDW, ACE, ADG, NS) independently searched the journal names for the eligible articles in InCites^TM^ Journal Citation Reports. No information was recorded for journals without a 2015 impact factor. We then entered article titles into the Scopus database search in order to locate the author profile for each corresponding author. When articles were not indexed in Scopus, corresponding authors’ first and last names were entered into the Scopus author search. For each corresponding author, we then identified the h-index and total number of publications. For each article published by the corresponding author included in our study, we used the full-text articles to determine the date of first publication (online or print). All uncertainties were discussed in detail, and a third reviewer (JSR) resolved any remaining discrepancies.

**Table 1:** Study design categories

Category	Examples
*Observational or other study*	Case-control studies, cross-sectional studies, cohort studies, retrospective analyses of data collected prospectively, case study/series, pharmacokinetic studies.
*Clinical trial*	Single or multi-arm prospective interventional studies with or without randomization.
*Systematic review and/or meta-analysis*	Any mention of the words “systematic review,” “systematic search,” “meta-analysis,” and “rapid systematic review” in the title and/or abstract.

In January 2017, we sent all potential survey respondents an email describing the purpose of the study, requesting their participation, and providing a link to the survey. Authors were informed that the survey should take less than 5 min. Four follow-up requests were sent by email over the course of February 2017. Although invitation to participate did not reference any specific study hypotheses, authors were informed that participation may provide information about the scientific process. Participation was completely voluntary and participants were informed of an opportunity to win one of five $100 gift certificates for Amazon. All internet-based responses were collected using a web-based survey platform (Qualtrics Labs, Provo, Utah, USA) [8].

### Survey instrument development

The design of our eight-item survey was informed by previously published surveys [6, 8], a review of the literature on post-submission experiences, and discussion among the authors (JDW, ACE, HMK, JSR). The instrument was pretested by researchers independent of the study team and modified iteratively to improve clarity, face validity, and content validity.

### Survey domains and dissemination

#### Perception of potential importance of manuscript

We asked authors to rate the potential importance (significance) of their manuscript as a contribution to the biomedical literature (1–5-point scale).

#### Submission history

We asked authors to select the approximate dates (month and year) when they first submitted the manuscript to any journal and when the manuscript was accepted for publication. We asked authors to specify (approximately): the total number of (1) journal submissions prior to acceptance, (2) journals that sent the manuscript for external peer review, (3) external peer reviewers that reviewed the manuscript across all submissions to all journals, and (4) journals that requested revisions to the manuscript.

#### Pre-print perceptions

We used a yes/no question to ascertain whether authors would have considered disseminating their research using a public online platform instead of submitting to a peer-reviewed journal to be published, assuming a situation in which sharing the manuscript online would provide the same amount of academic credit as a publication in a peer-reviewed journal.

### Statistical analysis

To compare characteristics of survey respondents and non-respondents, we used chi-squared tests for categorical variables and Wilcoxon-Mann-Whitney tests for continuous variables, using two-sided tests with a type I error level of 0.05. We then conducted descriptive analyses of the main respondent characteristics, journal details, and survey domains. Considering that the corresponding authors were asked to report the approximate month and year of first submission and acceptance, we recorded all dates as the first day of the corresponding month. After approximating the times from first submission to journal acceptance and publication, we removed all observations where there was evidence of potentially incorrect reporting (e.g., the time from first submission to journal acceptance was longer than the time from first submission to publication). Data were analyzed using SAS version 9.4 (SAS Institute, Cary, North Carolina, USA) and R (version 3.4.0: The R Project for Statistical Computing).

## Results

**Fig. 1:**
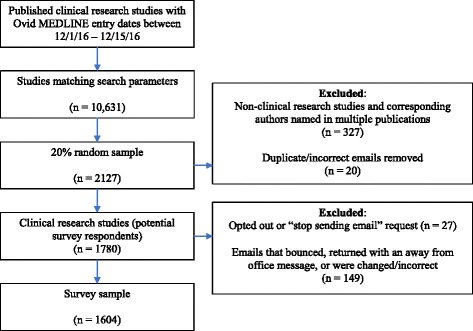
Flow chart showing identification and selection of potential articles

There were 1780 eligible corresponding authors who had published a clinical research article with Ovid MEDLINE entry dates between 1 December 2016 and 15 December 2016. Of the 1780 surveys distributed, 27 corresponding authors opted out or requested that we stop emailing them and 149 emails failed (e.g., emails that bounced *n* = 64, returned with an away from office message *n* = 70, or were changed/incorrect *n* = 15), leaving 1604 eligible respondents, of which 337 submitted a survey (response rate of 21.0%) (Fig. 1). All of the responding authors completed all of the survey questions (completion rate of 100.0%). Similar response rates were observed when we limited the sample to authors with the US articles or educational institution email addresses (e.g., universities or medical schools).

Survey respondents did not differ from non-respondents with respect to study type, publication journals’ impact factor, and journals’ country of publication. However, non-respondent corresponding authors had more publications (*p* = 0.03), slightly higher h-indices (*p* = 0.07), and were more likely to have non-institutional or Gmail/Yahoo/Hotmail/Aol email addresses (*p* = 0.003) (Table 2).

**Table 2:** Comparison of characteristics of survey respondents and non-respondents

	Respondents (*n* = 337) No. (%)	Non-respondents (*n* = 1443) No. (%)	*P* value
*Country of publication*			
USA	150 (44.5)	561 (38.9)	0.06
*Email*			
Institutional	232 (68.8)	1020 (70.7)	0.003
Gmail/Yahoo/Hotmail/Aol	80 (23.7)	248 (17.2)	
Other	25 (7.4)	175 (12.1)	
*Study design*			
Clinical trial	60 (17.8)	248 (17.2)	0.88
Observational or other study	247 (73.3)	1076 (74.6)	
Systematic review and/or meta-analysis	30 (8.9)	119 (8.3)	
*Corresponding author characteristics*			
Median number of publications (interquartile range)	27 (10.0 to 77.0)	38.0 (11.0 to 100.0)	0.03^a^
Median h-index (interquartile range)	9.0 (3.0 to 20.0)	11.0 (4.0 to 22.0)	0.07^a^
*Journal characteristics*			
Median journal impact factor (interquartile range)	2.7 (2.0 to 3.6)	2.6 (1.8 to 3.6)	0.31^a^

^a^Wilcoxon-Mann-Whitney test

### Article and corresponding author characteristics

**Fig. 2:**
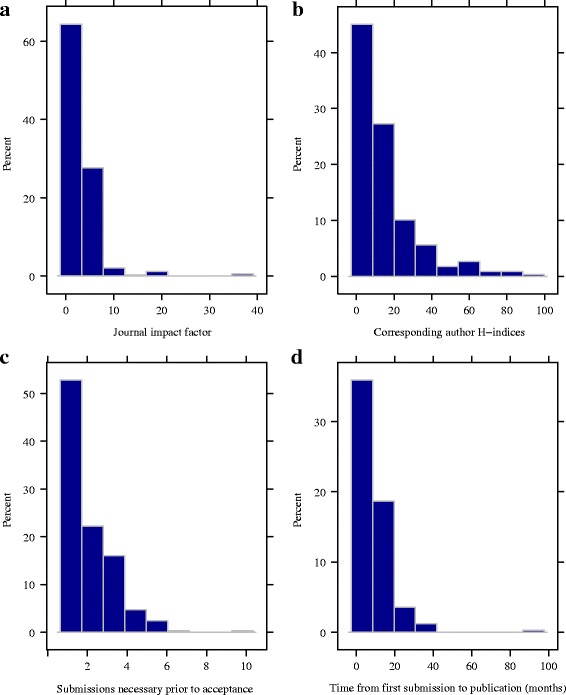
Histograms of (**a**) journal impact factor, (**b**) corresponding author h-indices, (**c**) submissions necessary prior to acceptances, and (**d**) time from first submission to publication (months)

Most respondents published observational studies (*n* = 247, 73.3%) in biomedical journals with a median impact factor of 2.7 (interquartile range (IQR), 2.0–3.6; Fig. 2a). Corresponding authors’ median h-index and number of publications was 9 (IQR, 3–20; Fig. 2b) and 27 (IQR, 10–77), respectively. Author and journal characteristics across study design categories are provided in (Table 3). A majority of respondents (62.0%, *n* = 209) rated the importance of their research as a 4 or 5 (on a 5-point scale) prior to first submission of the manuscript to any journal for consideration for publication.

**Table 3:** Characteristics of survey respondents across study design categories

	Clinical trials	Observational or other studies	Systematic reviews and/or meta-analyses
Median journal impact factor (interquartile range)	2.6 (2.1–3.6)	2.6 (1.9–3.5)	3.1 (2.5–4.0)
Median h-index (interquartile range)	11 (5–23)	9 (3–19)	11 (4–20)
Median number of publications (interquartile range)	32 (11–91)	27 (9–71)	37 (10–99)

### Journal and peer review experience

**Fig. 3:**
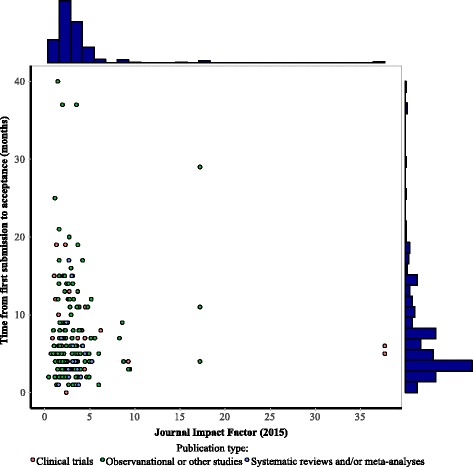
Time from first submission to acceptance (month) versus journal impact factor

Authors reported that anywhere between 1 and 10 submissions were necessary prior to acceptance (median 1 (IQR, 1–2), Fig. 2c). The median total number of journals that sent the manuscripts out for external peer review was 1 (IQR, 1–2), and the median total number of external peer reviewers that reviewed the manuscripts across all submissions to all journals was 3 (IQR, 2–4). The median time from first submission to journal acceptance and publication, among the 212 authors who were able to provide approximate months and years, was 5 months (IQR, 3–8; Fig. 3) and 7 months (IQR, 5–12; Fig. 2d), respectively. The median times from first submission to journal acceptances for clinical trials, observational or other studies, and systematic reviews and/or meta-analyses were 6 (IQR, 3–11), 5 (IQR, 3–8), and 4.0 (2–6), respectively. The median times from first submission to publication for clinical trials, observational or other studies, and systematic reviews and/or meta-analyses were 7 (IQR, 6–12), 7 (IQR, 5–12), and 6 (IQR, 5–9), respectively. Sharing manuscripts to an online platform, instead of submitting to a peer-reviewed journal, would have been considered by 55.2% of the corresponding authors (186 of 337).

## Discussion

In this pilot survey of corresponding authors who had recently published clinical research, we found that authors have high perceptions of their research. Authors reported requiring few manuscript submissions prior to journal acceptance, most commonly by lower impact factor journals. These findings suggest that the majority of clinical research studies in this sample were published within a year of first submission. Although our data come from a pilot study, our results may contradict prior perceptions that the biomedical publication process is too slow. Our results also suggest mixed perceptions about non-traditional publication practices.

In this feasibility study, we found that only 21% of potential participants completed our survey. This may suggest that an insufficient number of diverse corresponding authors of clinical research studies would respond to a large-scale online survey about the speed and efficiency of the broader publication process. Our response rate also does not compare favorably with other surveys of authors, physicians, and clinical trial investigators [1, 4, 6, 8]. Future cross-sectional surveys on this topic may benefit from focusing on specific groups of corresponding authors (e.g., those submitting from English-speaking countries or certain specialty groups). The results from this study are also likely subject to recall bias, including an inability of corresponding authors to remember specific details about a manuscript’s submission history. Considering that non-respondents had more publications than respondents, it is possible that non-respondents were unable to recall the submission history for one of their published articles. Furthermore, the automated emails requests could have been forwarded directly to the corresponding authors’ spam folders and language barriers may have limited the number of eligible respondents.

One possible explanation for our survey findings is that authors publishing clinical research articles are preferentially submitting their manuscripts to lower impact journals, possibly to ensure that articles do not require multiple submissions to multiple higher impact journals prior to eventual acceptance. Evidence suggests that the manuscripts selected for peer review in the highest impact journals can have extensive review times [9]. According to an empirical evaluation of articles submitted to the *Annals of Internal Medicine,* the majority of rejected manuscripts were published in lower impact factor specialty journals after an average of 18 months [10]. Our findings may reflect the fact that authors are willing to submit to just one lower impact journal in order to prevent publication delays that could result from multiple rejections and revisions at higher impact journals.

Technological advances could also be responsible for faster production times. According to an analysis of journal articles published in PubMed from 1960 to 2015, the median time that manuscripts spend in production is half of what it was in the early 2000s [9, 11]. Over the last few years, many journals have also started to embrace certain open science practices, including the sharing of *in press*, *ahead of print*, or *online first* articles, which could result in faster publication times. Journals may also be working to cut down on review times by increasing the number of papers that go through only one round of revision.

We found mixed perceptions when it comes to non-traditional publication practices. With the growing demand for faster dissemination of knowledge, journals as we know them may be “approaching their final act” [5]. However, publishing in journals remains the de facto currency for many academic investigators. Even though the corresponding authors were asked to imagine a situation where publicly sharing the manuscript online would provide the same amount of academic credit as a publication in a peer-reviewed journal, authors may find this a difficult scenario to envision, given the publish-or-perish atmosphere that exists at many academic institutions. Furthermore, clinical researchers may be fearful that competitive biomedical journals will not want to accept a manuscript that has already been made public.

### Limitations

First, only 21% of potential participants completed our survey. Second, the manuscript submission process and selection of a corresponding author may differ by institution, country, and research field. For example, a graduate student who worked on a project and submitted the manuscript likely would know more about the submission history than a senior investigator listed as the corresponding author. Based on the relatively low median h-index and number of publications, it is also possible that the majority of the respondents were early career researchers or less prolific investigators. As a result, our sample may not have sufficient diversity to draw generalizable conclusions. Furthermore, we found differences between respondents and non-respondents that suggest some response bias. In particular, non-respondents were more likely to have non-institutional email addresses and had more publications. These differences may suggest that the two groups differ from one another in more ways than they are similar and is a limitation of our study. Subsequent studies should explore these possibilities, ideally with larger survey samples that would permit multivariable analyses to account for potential differences between the respondents and non-respondents. Lastly, we used a non-validated survey instrument. However, we attempted to maximize ease of completion and limited the scope of the survey to reduce response burden.

## Conclusions

We found that corresponding authors have high perceptions of their research and reported requiring few manuscript submissions prior to journal acceptance, most commonly by lower impact factor journals. Furthermore, the majority of clinical research studies were published within a year of first submission, which suggests that the publication of clinical research studies is less delayed than was expected. These trends may explain why corresponding authors have mixed perceptions when it comes to non-traditional publication practices.

## Additional file

Additional file 1: I. Additional custom limits and specific search terms, II. Survey email, and III. Survey questions. (DOCX 129 kb)

## Abbreviations

CI: Confidence interval
IQR: Interquartile range
