# Supplementary material for: Biomedical journal speed and efficiency: a cross-sectional pilot survey of author experiences
Source: Res Integr Peer Rev. 2018 Jan 5;3:1. doi: 10.1186/s41073-017-0045-8 (PMC5803634; doi:10.1186/s41073-017-0045-8)
Supplement: Additional file 1: — I. Additional custom limits and specific search terms, II. Survey email, and III. Survey questions. (DOCX 129 kb) [file 41073_2017_45_MOESM1_ESM.docx]

**Supplementary Materials**

I. **Ovid search**

Ovid custom limits included: English language, humans, journal articles, and clinical research publications (*clinical study* or *clinical trial, all* or *clinical trial, phase I* or *clinical trial, phase ii* or *clinical trial, phase iii* or *clinical trial, phase iv* or *controlled clinical trial* or *meta analysis* or *observational study* or *pragmatic clinical trial* or *randomized controlled trial* or *systematic reviews*)).

Ovid search string:

(*cohort studies/ or longitudinal studies/ or follow-up studies or prospective studies/ or retrospective studies/ or cohort.ti,ab. or longitudinal.ti, ab. or prospective.ti, ab. or retrospective.ti, ab.*), clinical trials (*"clinical trial".pt. or "clinical trial, phase i".pt. or "clinical trial, phase ii".pt. or clinical trial, phase iii.pt. or clinical trial, phase iv.pt. or controlled clinical trial.pt. or "multicenter study".pt. or "randomized controlled trial".pt. or double-blind method/ or clinical trials as topic/ or clinical trials, phase i as topic/ or clinical trials, phase ii as topic/ or clinical trials, phase iii as topic/ or clinical trials, phase iv as topic/ or controlled clinical trials as topic/ or randomized controlled trials as topic/ or early termination of clinical trials as topic/ or multicenter studies as topic/ or ((randomized adj7 trial*) or (controlled adj3 trial*) or (clinical adj2 trial*) or ((single or doubl* or tripl* or treb*) and (blind* or mask*))).ti,ab,kw. or ("4 arm" or "four arm").ti,ab,kw.*), and systematic reviews or meta-analyses (*(((comprehensive* or integrative or systematic*) adj3 (bibliographic* or review* or literature)) or (meta-analy* or metaanaly* or "research synthesis" or ((information or data) adj3 synthesis) or (data adj2 extract*))).ti,ab. or (cinahl or (cochrane adj3 trial*) or embase or medline or psyclit or (psycinfo not "psycinfo database") or pubmed or scopus or "sociological abstracts" or "web of science").ab. or ("cochrane database of systematic reviews" or evidence report technology assessment or evidence report technology assessment summary).jn. or Evidence Report: Technology Assessment*.jn. or ((review adj5 (rationale or evidence)).ti,ab. and review.pt.) or meta-analysis as topic/ or Meta-Analysis.pt*). All of the terms and search strings were combined with “or”.

These search terms are from the University of Texas Health Sciences Center at Houston School of Public Health Library (Search Filters for Various Databases: Ovid Medline): <http://libguides.sph.uth.tmc.edu/search_filters/ovid_medline_filters)>

II. **Survey email:**

Dear Dr. ______ ,


My name is Joshua Wallach and I am a research fellow working with a group of investigators at Yale School of Medicine. We are conducting an 8 question survey (less than 5 minutes) of authors who recently published clinical research studies. The survey is intended to better understand the speed and efficiency of biomedical journal publication and how the process could be improved.
  
We would like to ask you to help us with this research by filling out this survey. Our questions are about your article ______ published in ______.

All participants will be automatically entered in a drawing to win one of five Amazon.com(c) gift cards worth $100. Participation in the survey is voluntary. Responses will remain strictly confidential and you will not be identified by your answers. Completion of the survey indicates consent to participate.

If you have any questions or concerns, please contact me ([joshua.wallach@yale.edu](mailto:joshua.wallach@yale.edu)).
 
Thank you for your participation!

**Follow this link to the Survey:**

Or copy and paste the URL below into your internet browser:

Joshua D. Wallach, MS, PhD
Research Fellow
Collaboration for Research Integrity and Transparency ([CRIT](https://medicine.yale.edu/core/current_projects/crit/))
Yale/YNHH Center for Outcomes Research & Evaluation ([CORE](https://medicine.yale.edu/core/))

Follow the link to opt out of future emails:

III. **Survey Questions**
1. Before you first submitted the manuscript to any journal for consideration for publication, on a scale from 1 to 5, how would you have rated its potential importance as a contribution to the biomedical literature? (i.e., how did you view the significance of the work at the time when first submitting the manuscript for publication).
2. On approximately what date did you first submit the manuscript to any journal for consideration for publication? (please select the month and year when the manuscript was first submitted to any journal, even if that journal did not accept the manuscript).
3. In total, to approximately how many journals did you submit the manuscript before it was accepted? (please select 1 if the manuscript was accepted by the first journal to which it was submitted).
4. In total, approximately how many of these journals sent the manuscript for external peer review? (please select a continuous value (i.e., 3) representing the total number of journals that solicited comments from peer reviewers); select 0 if the manuscript was accepted without peer-review).
5. In total, approximately how many external peer reviewers reviewed the manuscript across all submissions to all journals? (please select a continuous value (i.e., 3) representing the total number of peer reviewers; select 0 if the manuscript was accepted without peer-review).
6. In total, approximately how many of these journals requested revisions to the manuscript? (please select a continuous value (i.e., 3) representing the total number of peer reviewers; select 0 if the manuscript was accepted without peer-review).
7. On approximately what date (month and year) was your manuscript accepted for publication?
8. If an online platform existed where authors could share versions of their manuscript instead of submitting them to a journal to be published, would you have considered that as an option to disseminate the research described in this manuscript? (please imagine a situation where sharing the manuscript online would provide the same amount of academic credit as a publication in a peer-reviewed journal. Please select yes or no).
